# Characterization of Five Psychrotolerant *Alcanivorax* spp. Strains Isolated from Antarctica

**DOI:** 10.3390/microorganisms11010058

**Published:** 2022-12-24

**Authors:** Simone Cappello, Ilaria Corsi, Sabrina Patania, Elisa Bergami, Maurizio Azzaro, Monique Mancuso, Maria Genovese, Alessia Lunetta, Gabriella Caruso

**Affiliations:** 1 Institute for Biological Resources and Marine Biotechnologies, National Research Council (CNR-IRBIM), 98122 Messina, Italy; 2 Department of Physical, Earth and Environmental Sciences, University of Siena, 53100 Siena, Italy; 3 PhD School in “Applied Biology and Experimental Medicine”, University of Messina, 98166 Messina, Italy; 4 Department of Life Sciences, University of Modena and Reggio Emilia, 41125 Modena, Italy; 5 Institute of Polar Sciences, National Research Council (CNR-ISP), 98122 Messina, Italy

**Keywords:** *Alcanivorax* spp., Antarctica, molecular analyses, physiological analyses, biodegradation

## Abstract

Five psychrotolerant *Alcanivorax* spp. strains were isolated from Antarctic coastal waters. Strains were screened for molecular and physiological properties and analyzed regarding their growth capacity. Partial *16S rDNA*, *alk-B1*, and *P450* gene sequencing was performed. Biolog EcoPlates and the API 20E test were used to evaluate metabolic and biochemical profiles. Bacterial growth in sodium acetate was determined at 4, 15, 20, and 25 °C to evaluate the optimal temperature. Furthermore, the ability of each strain to grow in a hydrocarbon mixture at 4 and 25 °C was assayed. Biosurfactant production tests (drop-collapse and oil spreading) and emulsification activity tests (E_24_) were also performed. Concerning results of partial gene sequencing (*16S rDNA*, *alk-B1*, and *P450*), a high similarity of the isolates with the same genes isolated from other *Alcanivorax* spp. strains was observed. The metabolic profiles obtained by Biolog assays showed no significant differences in the isolates compared to the *Alcanivorax borkumensis* wild type. The results of biodegradative tests showed their capability to grow at different temperatures. All strains showed biosurfactant production and emulsification activity. Our findings underline the importance to proceed in the isolation and characterization of Antarctic hydrocarbon-degrading bacterial strains since their biotechnological and environmental applications could be useful even for pollution remediation in polar areas.

## 1. Introduction

Oil spills originating from shipping accidents, naval transports, or other causes represents one of the most serious threats affecting marine ecosystems worldwide [1]. Hydrocarbon pollution following these incidents causes highly negative effects on marine biodiversity on either plants or animals [2]; ecological consequences of oil spills are particularly serious in polar oceans, where low temperatures cause a slowdown of chemical, physical, and biological hydrocarbon degradation processes [3]. The first mechanisms activated after an oil spill are evaporation, dispersion, dissolution, emulsification, and photooxidation [4], followed by a microbial degradation step.

The existence of a particular group of marine hydrocarbon-degrading microorganisms, called obligate hydrocarbonoclastic bacteria (OHCB), is widely documented [5]; their main characteristic is a highly specialized metabolism to degrade hydrocarbons, and this allows them to play a predominant role in the biological removal of petroleum hydrocarbons from polluted marine waters. Several studies related to microbial hydrocarbon degradation were previously performed in cold marine environments [6,7,8] and psychrophilic alkane-degrading bacteria were isolated [9,10,11]; some of these strains were explored for their hydrocarbon-biodegradative potential [3,12].

The first identified strain belonging to the genus *Alcanivorax* was *A. borkumensis* [13], isolated near the Isle of Borkum (North Sea). Since then, more than 250 bacteria affiliated with the genus *Alcanivorax* have been isolated and/or detected [14] in different marine environments and in different geographical areas.

Bacteria related to the *Alcanivorax* genus are known to be able to degrade vast alkane hydrocarbons; the pivotal role in bioremediation activity played by *Alcanivorax* spp. was well documented [5,14]. The genetic basis of alkane degradation in *A. borkumensis* SK2 was also explored [15]. Particularly, this microorganism is capable of degrading large amounts of alkanes to C_32_ and branched aliphatics, as well as isoprenoid hydrocarbons (e.g., phytoene) and cycloalkane alkyl compounds. Degradation of alkanes in this species occurs through several terminal oxidation pathways involving alkB hydroxylase, flavin-binding monooxygenase, and cytochrome P450 [16].

Some oil-degrading bacteria belonging to the *Alcanivorax* genus were isolated from cold marine environments [10,17], and *alk* genes for alkane degradation described in *A. borkumensis* have been identified in Antarctic marine sediments [18]. In Antarctic soil, *16S rDNA* sequences phylogenetically related to strains *A. borkumensis* strain NJES-10 (accession no. KR140215, KR140213, KR140208) and operational taxonomic units (OTUs) affiliated with the *Alcanivorax* genus were found [19]. A new bacterial strain phylogenetically related to *Neptunomonas naphtovorans* (~95% of *16S rDNA* taxonomic identity) was isolated from Antarctic marine seawater and sediment [20]; the draft genome sequence of this new species, named *Neptunomonas antarctica* S3-22^T^, showed a total of 35 genes involved in the metabolism of aromatic hydrocarbon compounds [21]. To our knowledge, however, there are no reports regarding the isolation of bacterial strains related to *Alcanivorax* spp. from the Antarctic marine environment.

The present investigation focuses on the physiological and molecular characterization of five psychrotolerant *Alcanivorax* strains, isolated from a microbiological study of Antarctic surface seawater samples, with the aim to evaluate their metabolic differences and biotechnological potential.

## 2. Materials and Methods

### 2.1. Water Sampling and Treatment

Surface seawater samples were collected during the Antarctic summer of 2014–2015 from a site located in Terra Nova Bay (Antarctica), namely, the Road Bay (74°41.753′ S–164°07.188′ E) close to the Italian “Mario Zucchelli” scientific research station.

A 5 L Niskin bottle was used to collect seawater samples at 2 m below the ice pack through a 1.5 m diameter hole made by an ice drill. Within 2 h after sampling, cells (present in subaliquots samples, 1 L) were filtered through a 25 mm Nuclepore (0.2 μm pore size) black polycarbonate membrane (Costar, Pleasanton, CA, USA) and stored at 4 °C until laboratory analysis. An aliquot of seawater was used for bacterial enrichment.

### 2.2. Bacterial Enrichments and Isolation of Hydrocarbon-Degrading Strains

To set up bacterial enrichments, the seawater sample was filtered through a disposable vacuum filtration system (Millipore, Milan, Italy) and mixed in a 9/1 ratio (*v*/*v*) with ONR7a medium [22] supplemented with 0.5% (*w*/*v*) tetradecane (C_14_H_30_, Sigma-Aldrich, Milan, Italy) as the unique energy and carbon source. Bacterial inocula using a subsection of the previously obtained filter were performed. To allow the growth of oligotrophic bacteria, enrichments without tetradecane were carried out in parallel. Cultures were incubated at 4 ± 1 °C for 20 days. The growth of the bacterial assemblages was monitored by the development of turbidity (i.e., by optical density, OD, measurements at 600 nm, OD_600nm_) with a BioPhotometer (Eppendorf AG, Hamburg, Germany). After incubation, a set of serial dilutions from 10^−1^ to 10^−6^ were prepared by the initial culture enrichments. For each dilution, aliquots (100 µL) were directly spread onto duplicate agar plates of ONR7, a basal medium amended with tetradecane (0.1% *w*/*v*) as a single carbon source. The plates were incubated at 4 °C for 20 days. Among the grown colonies, those that had different phenotypic characteristics were isolated on plates of ONR7a with and without tetradecane to eliminate autotrophs and agar-utilizing bacteria. The plates were incubated under the same growth conditions as described above. All of the obtained isolates were spread on plates of Marine Agar (CondaLab, Torrejon de Ardoz, Spain) and ONR7a supplemented with sodium acetate (1%, *w*/*v*, Sigma-Aldrich, Milan, Italy) to determine the bacterial ability to grow on hydrocarbons as the sole carbon source.

### 2.3. Taxonomical Characterization of Hydrocarbon-Degrading Isolates

Genomic DNA was extracted from each isolated bacterial strain using the MasterPure™ Complete DNA and RNA Purification Kit (Epicentre, Illumina, San Diego, CA, USA). After elution with 35 μL of 1× TE buffer (pH 8), nucleic acids were quantified using a ND-1000 spectrophotometer (NanoDrop Technologies, Wilmington, DE, USA). *16S rRNA* gene sequences of the isolates were amplified from the genomic DNA using the universal eubacterial primer sets 27F-CM (5′-AGAGTTTGATCMTGGCTCAG-3′) and 1492R (5′-TACGGYTACCTTGTTACGACTT- 3′). PCR was performed using a 50 μL (total volume) mixture containing 1× Q solution (Qiagen, Hilden, Germany), 10× Qiagen reaction buffer, 1 μM of each primer, 10 μM dNTP (Gibco, Invitrogen, Carlsbad, CA, USA), 2.0 μL (40–250 ng) of DNA template, and 2.0 U of Taq Polymerase (Qiagen). The reaction started with a 3 min hot start at 95 °C, followed by 30 cycles of 1 min at 94 °C, 1 min at 50 °C and 2 min at 72 °C, and a final extension of 10 min at 72 °C. PCR products of the isolated strains were purified and sequenced (Sanger’s Method) by Macrogen Inc. (Amsterdam, The Netherlands) using only the reverse primer (1492R). *16S rRNA* gene sequences of the closest relatives were identified with the Basic Local Alignment Search Tool (BLAST), provided by the National Center for Biotechnology Information (NCBI, Bethesda, MA, USA) [23]. Sequences were submitted to the GenBank genetic sequence database at the NCBI; they are accessible with the following accession numbers: strain ANT5, with the accession number OP999659; strain ANT6, OP999660; strain ANT8, OP999661; strain ANT10, OP999662; strain ANT11, OP999663. After taxonomical characterization of all isolated strains, the analyses focused only on the five bacterial strains affiliated with the genus *Alcanivorax*.

### 2.4. Analyses on Alcanivorax spp. Strains

#### 2.4.1. Partial *16S rRNA*, *alk-B1*, and *P450* Gene Sequencing

Total DNA of *Alcanivorax* spp. strains was extracted and analysed using the MasterPure™ Complete DNA and RNA Purification Kit (Epicentre). After extraction, nucleic acids were quantified using an ND-1000 Spectrophotometer (NanoDrop Technologies). Amplification of *16S rRNA*, *alk-B1*, and *P450* genes was performed using the PCR protocol described above (see the 2.3 paragraph). Particularly, *alk-B1* and gene degenerate primers alk-BwF (5′–AAYACNGCNCAYGARCTNGGVCAYAA–3′ [24]) and alkBwR (5′–GCRTGRTGRTCHGARTGNCGYTG–3′ [24]) were used. The reaction started with a 4 min hot start at 94 °C, followed by 40 cycles of 30 sec at 94 °C, 30 sec at 55 °C and 1 min at 72 °C, and a final extension step of 10 min at 72 °C. For the *P450* gene, degenerate primers P450-F (5′-TGTCGGTTGAAATGTTCATYGCNMTGGAYC-3′) and P450-R (5′-TGCAGTTCGGCAAGGCGGTTDCCSRYRCAV-3′) were used. The reaction started with a 4 min hot start at 94 °C, continued with 40 cycles of 30 sec at 94 °C, 30 sec at 52 °C and 1 min at 72 °C, and finished with a 10 min extension at 72 °C. All amplified gene fragments were analysed on agarose gel (2% w/vol) and purified with a Wizard^®^ Genomic DNA Purification Kit (Promega, Madison, WI, USA). Amplified inserts of expected size (approx. 1200 bp for *16S rDNA*, 500 bp for *alk-B1*, and 800 bp for *P450* genes) were identified by gel electrophoresis. Sequencing was performed by the commercial services of Macrogen Inc. (Amsterdam, The Netherlands). Using the Molecular Evolutionary Genetics Analysis (MEGA 7) software (University of Kent, Canterbury, UK) [25], sequences obtained were aligned to *16S rRNA* closest relative’s sequences of the bacterial isolates present in the BLAST database [23]. After alignment, the neighbour-joining algorithm (1000 bootstrap) was used to generate the phylogenetic trees.

#### 2.4.2. Physiological Characterization

The bacterial isolates were tested for their ability to utilize different carbon sources using Biolog EcoPlates, according to the manufacturer’s instructions (Biolog, Inc., Hayward, CA, USA). Particularly, this miniaturized assay includes as carbon sources the following categories of substrates: carbohydrates, complex carbon sources, phosphate carbon sources, carboxylic and acetic acids, amino acids, and amines. Duplicate Biolog plates per each isolate were prepared and incubated at 4 ± 1 °C for 20 days. The colour development due to the reduction in the tetrazolium salt into formazan was analysed with the software package provided by Biolog Inc.

To characterize and identify the bacterial isolates, phenotypical tests (such as Gram staining and tests of mobility, oxidase, and catalase) were performed according to the *Bergey’s Manual of Systematic Bacteriology* (taxonomy) [26]. The biochemical profiles of the isolates were also evaluated by API 20E (bioMerieux, Marcy-l’Etoile, France) and the ability to grow on ONR7a supplemented with 1% (*v*/*w*) of sodium acetate and Marine Agar at 4 °C.

The growth of the five isolates at different temperatures was tested by preparing a starter culture of each strain in ONR7a supplemented with sodium acetate (1% *w*/*v*). Subsequently, 200 μL of mid-exponential phase cells was collected by centrifugation at 9000× *g* for 10 min according to the conventional protocols in use in our laboratory, washed twice with sterile medium, and inoculated into 250 mL sterile Erlenmeyer flasks, each containing 100 mL of ONR7a supplemented with 1% (*w*/*v*) sodium acetate. The strain cultures were incubated at 4, 15, 20, and 25 °C. Growth was monitored by OD_600_ measurements with a BioPhotometer (Eppendorf AG, Hamburg, Germany) approximately every 3 days for a total of 31 days.

The ability of the isolates to grow in a hydrocarbon mixture was evaluated by inoculating 200 μL of the same pre-inoculum used for the growth test at the temperature range (harvested by centrifugation at 9000× *g* for 10 min and washed twice with sterile medium) in 100 mL of ONR7a amended with 1% (*w*/*v*) crude oil (PierE1, Dansk Crude Oil) and incubating at 4 and 25 °C. Culture growth was monitored by OD_600 nm_ as indicated above.

#### 2.4.3. Evaluation of Biosurfactant Production and Emulsification Activity

During growth in ONR7a medium supplemented with PierE1 crude oil, biosurfactant production tests (drop-collapse and oil spreading tests) were performed on *Alcanivorax* isolates at 4 and 25 °C after 7 (T_7_) and 20 (T_20_) days from the beginning of the experiment, according to the protocol of Maneerat and Phetrong [27]. An emulsification activity test (E_24_) was carried out at T_7_ and T_20_ at the same experimental temperatures (4 and 25 °C) used for the biosurfactant production tests, with the method reported by Maia et al. [28]. Briefly, an equal volume of hexadecane and culture broth of each isolate was dispensed into a glass tube, then shaken for 2 min and left undisturbed for 24 h. The emulsification activity was defined as the height of the emulsified layer (mm) divided by the total height of the liquid column (mm) and expressed as a percentage [28].

## 3. Results

### 3.1. Isolation and Taxonomical Characterization of Hydrocarbon-Degrading Isolates

With the aim to identify Antarctic bacterial strains capable of degrading hydrocarbons, 43 strains were isolated from enrichment cultures in ONR7a medium supplemented with tetradecane as a single carbon source. Molecular characterization of isolates was carried out by amplifying and partially sequencing the *16S rRNA* gene (approx. 1200 bp); sequences were compared to the database of known *16S rRNA* sequences (BLAST). Identical gene sequences were grouped and only one representative isolate for each group was selected (Table 1). The phylogenetic analysis revealed the distribution of 13 selected isolates within the γ-Proteobacteria taxonomic group, and six different genera were detected. Particularly, six sequences were closely related to *Alcanivorax* spp.; fourteen sequences showed high similarity to the *Oleispira* genus (four *Oleispira antarctica* RB8 and ten *Oleispira lenta* DFH11). Twelve isolates had a phylogenetic affiliation with *Pseudoalteromonas* spp. and two sequences were related to *Marinomonas* sp. KJF11-23 and *Halomonas* sp. whb35, respectively. Finally, two sequences showed high similarity to *Vibrio artabrorum*. Table 1 also shows the results of growth tests of the isolates performed in a single ONR7a medium with and without the addition of sodium acetate as the carbon source and Marine Agar. All the strains were able to grow on ONR7a supplemented with sodium acetate; the strains a/ANT2a, a/ANT2d, a/ANT3, b/ANT9, b/ANT12a, and b/ANT15a were also able to grow on both ONR7a without sodium acetate and Marine Agar.

### 3.2. Analyses on Alcanivorax spp. Strains

From the phylogenetic analysis, five isolates, closely related to *Alcanivorax* spp. and indicated as a/ANT 5a, b/ANT 6, b/ANT 8, b/ANT 10, and b/ANT 11, were subject to further molecular and physiological analyses to better characterize them and to evaluate any differences with other bacteria related to the *Alcanivorax* genus.

### 3.3. Partial 16S rRNA, alk-B1, and P450 Gene Sequencing

**Figure 1:**
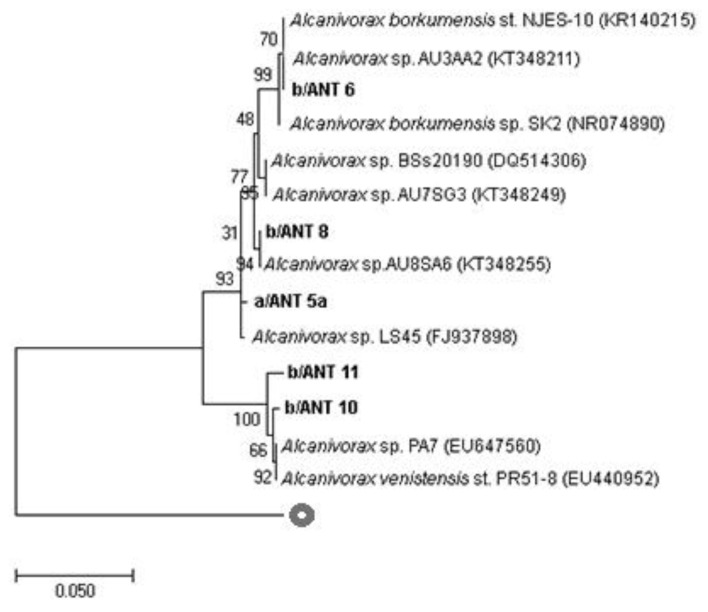
Rooted phylogenetic tree clustered by neighbour-joining of maximum likelihood values showing affiliation of complete bacterial *16S rRNA* gene sequences to closest related sequences from *Alcanivorax* spp. Isolates are indicated in bold-type. Percentages of 1000-bootstrap resampling that supported the branching orders in each analysis are shown above or near the relevant nodes. All trees were rooted and outgrouped with *16S rRNA* gene sequences of a *Bacillus subtilis* (AB192294). Vertical lines indicate evolutionary distance; each scale bar length corresponds to 0.05 fixed point mutations per sequence position.

For a more precise phylogenetic characterization of the *Alcanivorax* strains, partial *16S rRNA* gene sequencing was performed. Figure 1 shows the exact taxonomical position of the studied strains compared to the closest cultured relatives and other known *16S rRNA* sequences related to *Alcanivorax* spp. More in detail, a/ANT5a was closely related to *Alcanivorax* sp. LS 45 isolated from a marine sponge, *Gelliodes carnosa*, collected from coastal waters of the South China Sea [29]. b/ANT6 and b/ANT8 sequences revealed a phylogenetic affiliation with *Alcanivorax* sp. AU3AA2 and *Alcanivorax* sp. AU8SA6, respectively, isolated from a polluted marine coastal area in Priolo Bay, Sicily [30]. The last two isolates, b/ANT10 and b/ANT11, showed a high similarity with *Alcanivorax* sp. PA7 isolated from marine sediments in Argentina [31] and the *Alcanivorax venustensis* strain isolated from deep seawater on the Southwest Indian Ridge [32].

**Figure 2:**
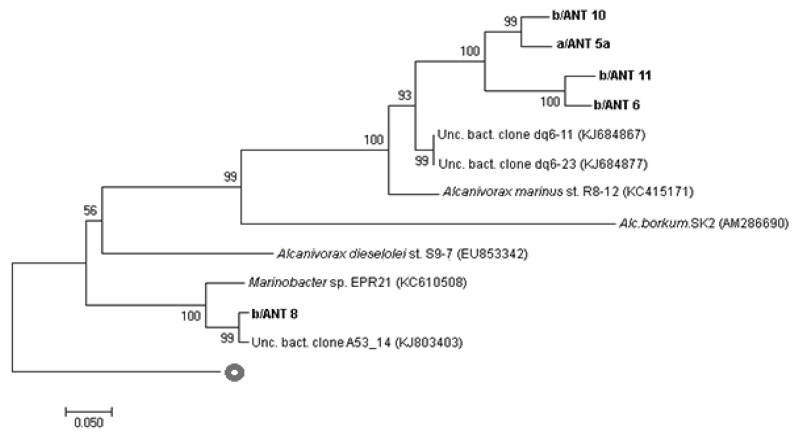
Rooted phylogenetic tree clustered by neighbour-joining of maximum likelihood values showing gene sequences to closest related sequences from *Alcanivorax* spp. Isolates are indicated in bold-type. Percentages of 1000-bootstrap resampling that supported the branching orders in each analysis are shown above or near the relevant nodes. All trees were rooted and outgrouped with *alk-B1* gene sequences of *Novosphingobium* sp. PCY *alkane monooxygenase* gene (KJ650249). Vertical lines indicate evolutionary distance; each scale bar length corresponds to 0.05 fixed point mutations per sequence position.

**Figure 3:**
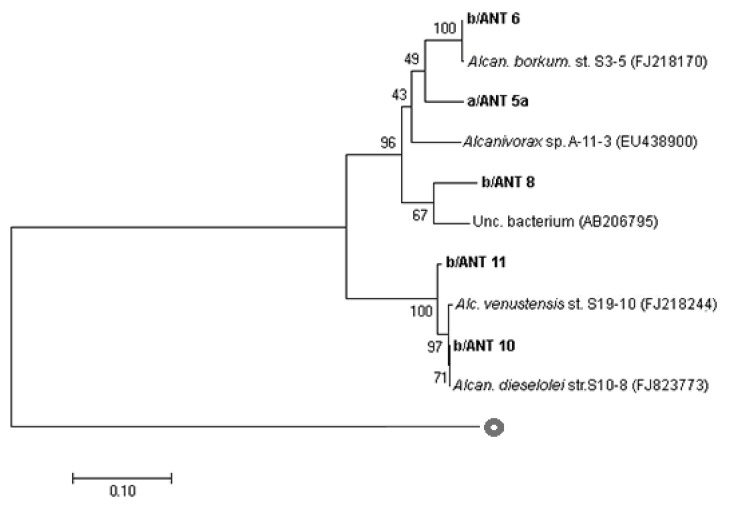
Rooted phylogenetic tree clustered by neighbour-joining of maximum likelihood values showing affiliation of *P450* gene sequences to closest related sequences from *Alcanivorax* spp. Isolates are indicated in bold-type. Percentages of 1000-bootstrap resampling that supported the branching orders in each analysis are shown above or near the relevant nodes. All trees were rooted and outgrouped with *P450* gene sequences of *Novosphingobium* sp. SB32149 gene for P450 (LC214353). Vertical lines indicate evolutionary distance; each scale bar length corresponds to 0.05 fixed point mutations per sequence position.

Gene sequencing of *alk-B1* and *P450* from *Alcanivorax* isolates was carried out to evaluate the degree of similarity with other known *alk-B1* and *P450* gene sequences related to *Alcanivorax* spp. (Figure 2 and Figure 3). Figure 2 shows the existence of a high similarity in the sequences of the *alk-B1* genes of the isolates a/ANT5a, b/ANT6, b/ANT10, and b/ANT11 to uncultured clones and to *Alcanivorax marinus* R8-12 (KC415171) isolated from deep sea water of the Indian Ocean [33]. The *alk-B1* gene sequence of the b/ANT 8 strain is poorly related to other analysed strains; it showed similarity with an uncultured clone and with *Marinobacter* EPR21 (KC610508).

*P450* gene sequences of isolates (Figure 3) are divided into two groups. Particularly, a/ANT5a, b/ANT6, and b/ANT8 sequences were related to *Alcanivorax borkumensis* S3-5 (FJ218170), and b/ANT10 and b/ANT11 sequences showed a high affiliation with *Alcanivorax dieselolei* S10-8 (FJ823773) and *Alcanivorax venustensis* S19-10 (FJ218244), respectively.

### 3.4. Physiological Characterization

The physiological profiles determined by Biolog EcoPlates showed that a/ANT5a, b/ANT6, and b/ANT8 strains exhibited only tweenase activity, while b/ANT10 and b/ANT11 strains showed a wider metabolic profile, with the capability of utilizing the following carbon sources: pyruvic acid, tween 40 and tween 80, D-cellobiose, erythritol, mannitol, N-acetyl-glucosamine, galacturonic acid, arginine, phenylalanine, threonine, and glycyl-glutamic acid.

Phenotypical tests indicated that all isolates were motile, Gram-negative, and showed oxidase, catalase, and gelatinase activities; b/ANT 10 and b/ANT 11 exhibited tryptophan deaminase activity and were positive for inositol and saccharose oxidation. Only b/ANT 10 was able to oxidize arabinose.

**Figure 4:**
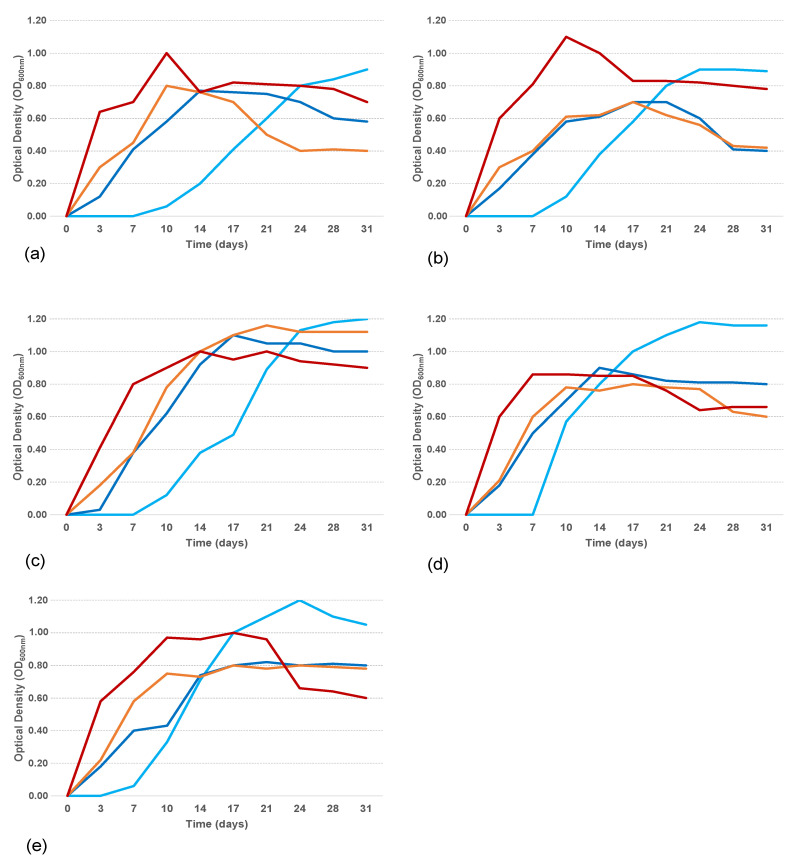
Growth curves at 4 (light blue line), 15 (blue line), 20 (orange line), and 25 °C (red line) for a/ANT5a (**a**), b/ANT6 (**b**), b/ANT8 (**c**), b/ANT10 (**d**), b/ANT11 (**e**) *Alcanivorax* strains in ONR7a medium amended with sodium acetate (1% *w*/*v*).

Growth curves in ONR7a supplemented with sodium acetate (1% *w*/*v*) at 4, 15, 20, and 25 °C showed that all the isolates grew actively at all tested temperatures (Figure 4).

**Figure 5:**
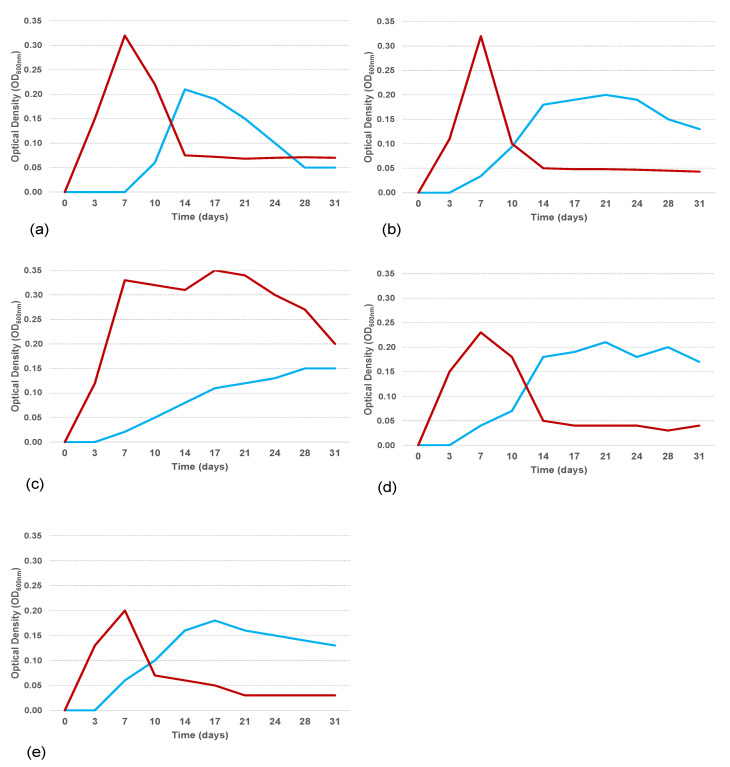
Growth curves at 4 °C (light blue line) and 25 °C (red line) for a/ANT5a (**a**), b/ANT6 (**b**), b/ANT8 (**c**), b/ANT10 (**d**), b/ANT11 (**e**) *Alcanivorax* strains in ONR7a medium amended with hydrocarbon mixture (PierE1, Dansk Crude Oil, 1% *w*/*v*).

At the end of the exponential growth phase, only the isolate b/ANT8 reached the same OD value regardless of the tested temperatures, while for the other isolates, the OD value obtained at 4 °C was higher than those measured at the other temperature values. Moreover, at a lower temperature, a longer stationary growth phase was observed and, consequently, exponential and stationary growth phases were delayed in time. The growth ability in the hydrocarbon mixture (PierE1, Dansk Crude Oil, 1% *w*/*v*) of *Alcanivorax* strains was tested at 4 and 25 °C (Figure 5). For all the strains, during cultivation at 25 °C the biomass increased during the first 17 days of experimentation; conversely, during cultivation at 4 °C the dynamic of growth was slower with an extended lag phase. After day 17 this trend was inverted, with a biomass increase at 4 °C and a reduction at 25 °C. The behaviour of the growth curves likely depended on the biodegradation processes and therefore on the use of the contaminant as a source for growth.

OD measures demonstrated that all isolates showed a different growth capability at the two temperatures, 4 and 25 °C. Particularly, at 25 °C, all isolates reached the end of the exponential phase after 7 days; a/ANT5a, b/ANT6, and b/ANT8 strains showed the highest values of approx. 0.3 OD_600nm_, compared to the b/ANT10 and b/ANT11 strains whose OD reached values of approximately 0.2. At 4 °C, a longer lag phase was detected in all strains, and lower OD values at the end of the exponential phase, compared to those observed at 25 °C, except for the strains b/ANT10 and b/ANT11 that showed the same OD values (equal to 0.2) at both growth temperatures.

### 3.5. Biosurfactant Production and Emulsification Activity

Drop-collapse and oil spreading methods were performed to analyse biosurfactant production of the studied strains. In addition, an emulsification activity assay was tested for each strain. All tests were carried out at 4 and 25 °C and after 7 (T_7_) and 20 (T_20_) days post-inoculation, given the decreased growth rate of the strains at lower temperatures. The drop-collapse test showed negative results (no emulsifier halo) for all studied strains at 4 and 25 °C, both at T_7_ and T_20_ (Table 2).

The oil spreading test showed that all the strains at both temperatures showed positive results after 7 days (T_7_) and 20 days (T_20_). The maximum value of 2.0 ± 0.1 cm was measured for the isolate a/ANT5a at T_7_ (25 °C) and a minimum of 0.2 ± 0.1 cm for the isolates a/ANT5a and b/ANT10 at T_20_ (4 and 25 °C, respectively). Particularly, a clear reduction in the formation of the halo (less than 1 cm) through time was recorded for the a/ANT5a, b/ANT6, b/ANT10, and b/ANT11 isolates; no halo formation was detected for the b/ANT8 isolate after 20 days at both 4 and 25 °C.

The emulsification activity assay (E_24_) showed no emulsion formation at 4 °C in all strains, except for the b/ANT6 isolate at T_20_ (14.3 ± 0.5). Conversely, all the strains showed emulsification activity at 25 °C, which decreased remarkably after 20 days.

## 4. Discussion

Bacterial strains belonging to the *Alcanivorax* genus were previously isolated from cold marine environments [10] and the presence of *alk* genes was detected in Antarctica [18]. The present study describes hydrocarbon-degrading bacterial strains closely related to the *Alcanivorax* genus isolated from an Antarctic marine environment. Particularly, a total of 43 bacterial strains were isolated through enrichment cultures from an area of the Ross Sea (Antarctica) close to the Italian scientific research station and phylogenetically analysed. Subsequently, attention was focused on five strains phylogenetically related to the *Alcanivorax* genus, considering their importance, which were subject to physiological and phylogenetic analyses, biosurfactant production tests, and emulsification activity assays.

The interest addressed to these strains is justified by the fact that, although they are taxonomically related to a known genus, they were selected in a cold environment; therefore, the presence of adaptation strategies comparable to *Alcanivorax* SK2^T^ [8] can be hypothesized (e.g., production of cold-shock proteins and/or activation of alternative metabolic pathways) [34].

Complete *16S rRNA*, *alk-B1*, and *P450* gene sequencing of Ross Sea surface waters isolated strains showed a phylogenetic affiliation with *Alcanivorax* spp. isolated from non-cold environments; this could explain the psychrotolerance of these strains, which are able to grow at higher temperatures than those present in the Antarctic seawaters (mean surface seawater temperature in Terra Nova Bay, the area where samples for this study were collected, is about −2 °C). From the carbon substrate utilization profiles obtained by Biolog EcoPlates, no differences were detected between a/ANT5a, b/ANT6, and b/ANT8 strains and *A. borkumensis* SK2^T^ [8], exhibiting only tween-degrading activity. Growth curves obtained in culture media with both sodium acetate and oil showed that the studied strains were able to grow at all tested temperatures; however, lower temperatures were associated with lower growth rates, and a lower cellular abundance with a longer lag phase was observed when the isolates were cultivated in a medium containing oil as the carbon source. *The surfactant obtained from Alcanivorax borkumensis* SK2 was suggested as one of the most effective bacterial surfactants [15,17].

According to Yakimov et al. [5], all strains exhibited extracellular surfactant production as evidenced by oil spreading but not by the drop-collapse test. The discrepancy between these results may suggest that there was a production of biosurfactants (as found by the oil spreading test) but at low concentrations only. In fact, in the drop-collapse test, the stability of oil drops in the wells is dependent on biosurfactant concentration, and the presence of scarce amounts of biosurfactant produces negative results. Regardless, the possible presence of this biosurfactant can justify an increase in the rates of degradation [15,17].

Isolation of hydrocarbon-degrading bacteria from Antarctic soils has often been reported [19,35,36]. Sequences related to oil-degrading bacteria of the genera *Alcanivorax* and *Marinobacter* were also detected within the prokaryotic community colonizing some plastic fragments retrieved from the sub-Antarctic area of King George Island and the potential role of these microorganisms as plastic degraders was suggested [37]. Conversely, studies regarding the occurrence and ecology of OHCB in Antarctic seawater are quite scarce [38]; consequently, the present study confirms the presence of psychrotolerant hydrocarbon-degrading bacteria in Antarctic surface waters, widening knowledge about their ecophysiology.

Microbial degradation of petroleum hydrocarbons is limited by low temperatures. At low temperatures, the oil viscosity is high and sea ice is considered impermeable. In these nutrient-deficient environmental conditions, oil biodegradation is restricted to the small contact area between ice-inhabiting oil-degrading bacteria. In any case, the presence of hydrocarbon-degrading bacteria in the microbial community structure is directly related to the chemical composition of the pollutants (e.g., the different hydrocarbons present in the oil); on the other hand, the occurrence of these bacteria has considerable potential implications in terms of the biodegradation of oil compounds.

Dastgheib et al. [39] also stated that the optimal growth temperature of an *Alcanivorax strain* was about 35 °C. Similarly, Liu et al. [40] isolated a mesophilic *Alcanivorax* strain that used octadecane as the sole carbon source and its optimal growth temperature was within 30–37 °C. Moreover, Golyshin et al. [41] showed that the optimal temperature for growth of *Alcanivorax* spp. is between 25 and 30 °C. According to Morita [42], all bacteria isolated were not psychrophiles, but rather psychrotolerant, with optimum temperatures >20 °C but capable of growing at/or near 0 °C. Given the temperature up to 20 °C that Antarctic surface soils may reach in summer [43], the classification of these bacteria as psychrotolerant appeared to be appropriate.

Psychrotolerant bacteria, which are adapted to a wider temperature range, may have important advantages in biotechnological applications and have a high scientific relevance, especially for environmental monitoring and the safeguarding of these extreme ecosystems from anthropic impacts.

The study of genome sequence and functional genomic analysis of the oil-degrading bacterium *Oleispira antarctica* [44] and *A. borkumensis* [15] gives important information about the mechanism of hydrocarbon degradation.

Alkanes of lower molecular weight tend to degrade faster than those of greater molecular weight due to presence of shorter carbon chains. Degradation of short-chain alkanes with Antarctic bacteria was reported [45]. Alkanes ranging from C_9_ to C_14_ are the most detected in Antarctic soil, and this aliphatic fraction represents the major source of pollution near Antarctic stations. Thus, microbial populations with the competence to degrade C_12_–C_18_ are needed for the biodegradation and bioremoval of oil spillages around Antarctic research stations, as diesel oil typically consists of n-alkanes in this range. Whyte et al. [46] reported the detection and characterization of alkane-degrading (*alk-B* and *alk-M*) genotypes in both hydrocarbon-contaminated and not-contaminated Antarctic soils.

The phylogenetic characterization of Antarctic hydrocarbon-degrading bacteria assumes a particular importance from both a purely biotechnological and an environmental point of view, considering the potential application of these microorganisms for the recovery of contaminated polar areas.

## Figures and Tables

**Table 1:** Phylogenetic relationships of the examined bacterial isolates and results of growth tests performed on different solid culture media.

Selected Isolate	No. of Identical Sequences	Closest Species	GenBank No.	Sequence Similarity (%)	Sequence Size (bp)	ONR7a	ONR7a/ Na-Acetate	Marine Agar
*Alcanivorax*								
a/ANT5a	1	*Alcanivorax* sp. LS45	FJ937898	99	670	−	+	−
b/ANT6	2	*Alcanivorax* sp. AU3AA2	KT348211	100	721	−	+	−
b/ANT8	1	*Alcanivorax* sp. AU8SA6	KT348255	99	560	−	+	−
b/ANT10	1	*Alcanivorax* sp. PA7	EU647560	99	632	−	+	−
b/ANT11	1	*A. venustensis* strain PR51-8	EU440952	99	650	−	+	−
*Oleispira*								
a/ANT1a	4	*Oleispira antarctica* RB8	FO203512	99	634	−	−	−
a/ANT4a	10	*Oleispira lenta* strain. DFH11	NR108293	99	534	−	−	−
*Pseudoalteromonas*								
a/ANT2a	6	*Pseudoalteromonas* sp. 204Z-3	GU584162	99	623	+	+	+
a/ANT2d	5	*Pseudoalteromonas* sp. KJF2-15	JQ800018	100	660	+	+	+
a/ANT3	1	*Pseudoalteromonas* sp. H2-49	KM979170	99	701	+	+	+
*Marinomonas*								
b/ANT9	1	*Marinomonas* sp. KJF11-23	JQ800192	99	637	+	+	+
*Halomonas*								
b/ANT12a	8	*Halomonas* sp. whb35	FJ444981	100	739	+	+	+
*Vibrio*								
b/ANT15a	2	*Vibrio artabrorum*	FN667877	99	732	+	+	+

Number of identical sequences, No. of identical sequences; GenBank accession number, GenBank No.; bp, base pairs.

**Table 2:** Biosurfactant production (by Drop-collapse and oil spreading tests) and emulsification activity (%, E24) of the studied strains found at 4 and 25 °C and after 7 (T7) and 20 (T20) days post-inoculation.

	Isolate	Drop-Collapse		Oil Spreading (cm)		E_24_ Test (%)	
		T_7_	T_20_	T_7_	T_20_	T_7_	T_20_
4 °C	a/ANT5a	-	-	1.0 ± 0.1	0.2 ± 0.1	-	-
	b/ANT6	-	-	1.5 ± 0.1	0.4 ± 0.1	-	14.3 ± 0.5
	b/ANT8	-	-	1.0 ± 0.1	-	-	-
	b/ANT10	-	-	0.8 ± 0.1	0.3 ± 0.1	-	-
	b/ANT11	-	-	1.0 ± 0.1	0.4 ± 0.1	-	-
25 °C	a/ANT5a	-	-	2.0 ± 0.1	0.2 ± 0.1	58.6 ± 0.5	15.5 ± 0.5
	b/ANT6	-	-	1.9 ± 0.1	0.3 ± 0.1	75.2 ± 0.5	27.6 ± 0.5
	b/ANT8	-	-	1.0 ± 0.1	-	62.6 ± 0.5	3.4 ± 0.5
/	b/ANT10	-	-	1.0 ± 0.1	0.2 ± 0.1	60.0 ± 0.5	3.4 ± 0.5
	b/ANT11	-	-	0.7 ± 0.1	0.5 ± 0.1	5.5 ± 0.5	3.4 ± 0.5

## Data Availability

Data are available upon request.

## References

[1] Fingas M. (2012). The Basics of Oil Spill Cleanup.

[2] Xue J., Yu Y., Bai Y., Wang L., Wu Y. (2015). Marine Oil-Degrading Microorganisms and Biodegradation Process of Petroleum Hydrocarbon in Marine Environments: A Review. Curr. Microbiol..

[3] Michaud L., Lo Giudice A., Saitta M., De Domenico M., Bruni V. (2004). The biodegradation efficiency on diesel oil by two psychrotrophic Antarctic marine bacteria during a two-month-long experiment. Mar. Pollut. Bull..

[4] Radovic’ J.R., Aeppli C., Nelson R.K., Jimenez N., Reddy C.M., Bayona J.M., Albaigés J. (2014). Assessment of photochemical processes in marine oil spill fingerprinting. Mar. Pollut. Bull..

[5] Yakimov M.M., Timmis K.N., Golyshin P.N. (2007). Obligate oil-degrading marine bacteria. Curr. Opin. Biotechnol..

[6] Hazen T.C., Dubinsky E.A., DeSantis T.Z., Andersen G.L., Piceno Y.M., Singh N., Jansson J.K., Probst A., Borglin S.E., Fortney J.L. (2010). Deep-sea oil plume enriches indigenous oil-degrading bacteria. Science.

[7] Guibert L.M., Loviso C.L., Marcos M.S., Commendatore M.G., Dionisi H.M., Lozada M. (2012). Alkane Biodegradation Genes from Chronically Polluted Subantarctic Coastal Sediments and Their Shifts in Response to Oil Exposure. Microb. Ecol..

[8] Yakimov M.M., Giuliano L., Gentile G., Crisafi E., Chernikova T.N., Abraham W.R., Lunsdorf H., Timmis K.N., Golyshin P.N. (2003). *Oleispira antarctica* gen. nov., sp. nov., a novel hydrocarbonoclastic marine bacterium isolated from Antarctic coastal seawater. Int. J. Syst. Evol. Microbiol..

[9] Gentile G., Bonasera V., Amico C., Giuliano L., Yakimov M. (2003). *Shewanella* sp. GA-22, a psychrophilic hydrocarbonoclastic Antarctic bacterium producing polyunsaturated fatty acids. J. Appl. Microbiol..

[10] Cai Q., Zhang B., Chen B., Zhu Z., Lin W., Cao T. (2014). Screening of biosurfactant producers from petroleum hydrocarbon contaminated sources in cold marine environments. Mar. Pollut. Bull..

[11] Jain D.K., Collins-Thompson D.L., Lee H., Trevors J.T. (1991). A drop-collapsing test for screening surfactant-producing microorganisms. J. Microbiol. Meth..

[12] Gentile G., Bonsignore M., Santisi S., Catalfamo M., Giuliano L., Genovese L., Yakimov M.M., Denaro R., Genovese M., Cappello S. (2016). Biodegradation potentiality of psychrophilic bacterial strain *Oleispira antarctica* RB-8^T^. Mar. Pollut. Bull..

[13] Silveira C.B., Thompson F., Rosenberg E., DeLong E.F., Lory S., Stackebrandt E., Thompson F.. (2014). The family *Alcanivoraceae*. The Prokaryotes—Gammaproteobacteria.

[14] Crisafi F., Giuliano L., Yakimov M.M., Azzaro M., Denaro R. (2016). Isolation and degradation potential of a cold-adapted oil/PAH-degrading marine bacterial consortium from Kongsfjorden (Arctic region). Rend. Lincei-Sci. Fis..

[15] Schneiker S., Dos Santos V.A., Bartels D., Bekel T., Brecht M., Buhrmester J., Chernikova T.N., Denaro R., Ferrer M., Gertler C. (2006). Genome sequence of the ubiquitous hydrocarbon-degrading marine bacterium *Alcanivorax borkumensis*. Nat. Biotechnol..

[16] Sabirova J.S., Becker A., Lunsdorf H., Nicaud J.M., Timmis K.N., Golyshin P.N. (2011). Transcriptional profiling of the marine oil-degrading bacterium *Alcanivorax borkumensis* during growth on n-alkanes. FEMS Microbiol. Lett..

[17] Yakimov M.M., Golyshin P.N., Lang S., Moore E.R.B., Abraham W.R., Lünsdorf H., Timmis K.N. (1998). *Alcanivorax borkumensis* gen. now, sp. nov., a new, hydrocarbon-degrading and surfactant-producing marine bacterium. Int. J. Syst. Evol. Microbiol..

[18] Kuhn E., Bellicanta G.S., Pellizari V.H. (2009). New *alk* genes detected in Antarctic marine sediments. Environ. Microbiol..

[19] Goordial J., Davila A., Lacelle D., Pollard W., Marinova M.M., Greer C.W., DiRuggiero J., McKay C.P., Whyte L.G. (2016). Nearing the cold-arid limits of microbial life in permafrost of an upper dry valley, Antarctica. ISME J..

[20] Dyksterhouse S.E., Gray J.P., Herwig R.P., Lara J.C., Staley J.T. (1995). *Cycloclasticus pugetii* gen. nov., sp. nov., an Aromatic Hydrocarbon-Degrading Bacterium from Marine Sediments. Int. J. Syst. Evol. Microbiol..

[21] Altschul S.F., Gish W., Miller W., Myers E.W., Lipman D.J. (1990). Basic local alignment search tool. J. Mol. Biol..

[22] Zhang X.Y., Zhang Y.J., Yu Y., Li H.J., Gao Z.M., Chen X.L., Chen B., Zhang Y.Z. (2010). *Neptunomonas antarctica* sp. nov., isolated from marine sediment. Int. J. Syst. Evol. Microbiol..

[23] Rong J.C., Liu M., Li Y., Sun T.Y., Xie B.B., Shi M., Chen X.L., Qin Q.L. (2016). Insight into the genome sequence of a sediment-adapted marine bacterium *Neptunomonas antarctica* S3-22^T^ from Antarctica. Mar. Genom..

[24] Hou D., Shi Z., Shen X., He Y., Sun M., Iuo Q., Wang Q. (2013). Isolation, identification and alkane hydroxylase genes detection of a marine diesel-degrading bacterial strain (F9). Afr. J. Microbiol. Res..

[25] Kumar S., Stecher G., Tamura K. (2016). Molecular Evolutionary Genetics Analysis version 7.0 for big datasets. Mol. Biol. Evol..

[26] Holt S.G., Krieg N.R., Sneath P.H.A., Stanley J.T., Williams S.T. (1998). Bergey’s Manual of Determinate Bacteriology.

[27] Maneerat S., Phetrong K. (2007). Isolation of biosurfactant-producing marine bacteria and characteristics of selected biosurfactant. J. Sci. Technol..

[28] Maia M., Capão A., Procópio L. (2019). Biosurfactant produced by oil-degrading *Pseudomonas putida* AM-b1 strain with potential for microbial enhanced oil recovery. Bioremediat. J..

[29] Li C.Q., Liu W.C., Zhu P., Yang J.L., Cheng K.D. (2011). Phylogenetic Diversity of Bacteria Associated with the Marine Sponge *Gelliodes carnosa* collected from the Hainan Island Coastal Waters of the South China Sea. Microb. Ecol..

[30] Catania V., Santisi S., Signa G., Vizzini S., Mazzola A., Cappello S., Yakimov M.M., Quatrini P. (2015). Intrinsic bioremediation potential of a chronically polluted marine coastal area. Mar. Pollut. Bull..

[31] Olivera N.L., Nievas M.L., Lozada M., del Prado G., Dionisi H.M., Faustino Siñeriz F. (2009). Isolation and characterization of biosurfactant-producing *Alcanivorax* strains: Hydrocarbon accession strategies and alkane hydroxylase gene analysis. Res. Microbiol..

[32] Yuan J., Lai Q., Sun F., Zheng T., Shao Z. (2015). The diversity of PAH-degrading bacteria in a deep-seawater column above the Southwest Indian Ridge. Front. Microbiol..

[33] Lai Q., Wang J., Gu L., Zheng T., Shao Z. (2013). *Alcanivorax marinus* sp. nov., isolated from deep-sea water. Int. J. Syst. Evol. Microbiol..

[34] Park C., Park W. (2018). Survival and Energy Producing Strategies of Alkane Degraders Under Extreme Conditions and Their Biotechnological Potential. Front. Microbiol..

[35] Gran-Scheuch A., Fuentes E., Bravo D.M., Jiménez J.C., Pérez-Donoso J.M. (2017). Isolation and Characterization of Phenanthrene Degrading Bacteria from Diesel Fuel-Contaminated Antarctic Soils. Front. Microbiol..

[36] Aislabie J., Foght J., Saul D. (2000). Aromatic hydrocarbon-degrading bacteria from soil near Scott Base, Antarctica. Polar Biol..

[37] Cappello S., Caruso G., Bergami E., Macrì A., Venuti V., Majolino D., Corsi I. (2021). New insights into the structure and function of the prokaryotic communities colonizing plastic debris collected in King George Island (Antarctica): Preliminary observations from two plastic fragments. J. Hazard. Mater..

[38] Zappalà G., Caruso G., Denaro R., Crisafi F., Monticelli L.S. (2020). Ecological and molecular approach to the assessment of oil pollution: A comparative study between two coastal marine (Mediterranean and Patagonian) ecoregions. WIT Trans. Ecol. Environ..

[39] Dastgheib S.M.M., Amoozegar M.A., Khajeh K., Ventosa A. (2011). A halotolerant *Alcanivorax* sp. strain with potential application in saline soil remediation. Appl. Microbiol. Biotechnol..

[40] Liu Y.C., Li L.Z., Wu Y., Tian W., Zhang L.P., Xu L., Shen Q.R., Shen B. (2010). Isolation of an alkane-degrading *Alcanivorax* sp. strain 2B5 and cloning of the alkB gene. Bioresour. Technol..

[41] Golyshin P.N., Harayama S., Timmis K.N., Yakimov M.M. (2007). Family II. Alcanivoraceae fam. nov. Bergey’s Manual of Systematic Bacteriology: Volume 2: The Proteobacteria, Part B: The Gammaproteobacteria.

[42] Morita R.Y. (1975). Psychrophilic bacteria. Bacteriol. Rev..

[43] Campbell I.B., Claridge G.G.C., Campbell D.I., Balks M.R., Priscu J.C. (1998). The soil environment of the McMurdo Dry Valleys, Antarctica. Ecosystem Dynamics in a Polar Desert. The McMurdo Dry Valleys, Antarctica.

[44] Kube M., Chernikova T.N., Al-Ramahi Y., Beloqui A., Lopez-Cortez N., Guazzaroni M.E., Heipieper H.J., Klages S., Kotsyurbenko O.R., Langer I. (2013). Genome sequence and functional genomic analysis of the oil-degrading bacterium *Oleispira antarctica*. Nat. Commun..

[45] Habib S., Ahmad S.A., Johari W.L., Shukor M.Y., Alias S.A., Khalil K.A., Yasid N.A. (2018). Evaluation of conventional and response surface level optimisation of *n*-dodecane (*n*-C_12_) mineralisation by psychrotolerant strains isolated from pristine soil at Southern Victoria Island, Antarctica. Microb. Cell Factories.

[46] Whyte L.G., Schultz A., van Beilen J.B., Luz A.P., Pellizari V., Labbé D., Greer C.W. (2002). Prevalence of alkane monooxygenase genes in Arctic and Antarctic hydrocarbon-contaminated and pristine soils. FEMS Microbiol. Ecol..

